# The altered Granger causality connection among pain-related brain networks in migraine

**DOI:** 10.1097/MD.0000000000010102

**Published:** 2018-03-09

**Authors:** Yanzhe Ning, Ruwen Zheng, Kuangshi Li, Yong Zhang, Diyang Lyu, Hongxiao Jia, Yi Ren, Yihuai Zou

**Affiliations:** a Department of Neurology and Stroke Center, Dongzhimen Hospital, the First Affiliated Hospital of Beijing University of Chinese Medicine; b The National Clinical Research Center for Mental Disorders & Beijing Key Laboratory of Mental Disorders, Beijing Anding Hospital, Capital Medical University; c Department of Acupuncture and Moxibustion, Dongfang Hospital, The Second Affiliated Hospital of Beijing University of Chinese Medicine; d Department of Internal Medicine, Gulou Hospital of Traditional Chinese Medicine of Beijing.

**Keywords:** Granger causality analysis, migraine, pain-related networks, resting-state functional magnetic resonance imaging

## Abstract

Numerous fMRI studies have confirmed functional abnormalities in resting-state brain networks in migraine patients. However, few studies focusing on causal relationships of pain-related brain networks in migraine have been conducted. This study aims to explore the difference of Granger causality connection among pain-related brain networks in migraine without aura (MWoA) patients.

Twenty two MWoA patients and 17 matched healthy subjects were recruited to undergo resting-state fMRI scanning. Independent component analysis was used to extract pain-related brain networks, and Granger causality analysis to characterize the difference of Granger causality connection among pain-related brain networks was employed.

Seven pain-related brain networks were identified, and MwoA patients showed more complex Granger causality connections in comparison with healthy subjects. Two-sample t test results displayed that there was the significant difference between right-frontoparietal network (RFPN) and executive control network (ECN).

This study indicates that the specific intrinsic brain Granger causality connectivity among pain-related networks in MwoA patients are affected after long-term migraine attacks.

## 1 Introduction

Migraine, a chronic neurological disorder, has brought great attention from the public due to its high prevalence and large medical burden, according to the Global Burden of Disease (GBD 2010).^[1,2]^ For migraine patients, the quality of daily life is seriously influenced by the repeated migraine attacks and its ubiquitous sleep disorders. For children, attachment styles, maternal personality profile, and motor coordination impairment are affected by migraine.^[3–5]^ Thus, to study the neural mechanisms of migraine is of great importance.

In the past decades, with the fast development of functional magnetic resonance imaging (fMRI), it has opened a window to explore the pathogenesis of neurological disorders. Significant improvements have been made in the field of researches on the mechanisms of diseases, such as stroke,^[6]^ Alzheimer's disease,^[7]^ depression,^[8]^ knee osteoarthritis,^[9]^ and so on. Recently, it has also been applied in migraine to explore the changes of intrinsic brain activity after long-term pain attacks.^[10,11]^ In the aspect of whole brain, amplitude of low frequency fluctuations and graph theory were used to investigate the change of spontaneous neural activity after long-term pain attacks.^[12,13]^ As for regional resting-state networks, previous studies demonstrated that migraine without aura (MwoA) patients had the abnormal functional connectivity within the default mode network (DMN),^[14]^ executive control network (ECN),^[15]^ sensorimotor network (SMN),^[16]^salience network (SN),^[17,18]^ periaqueductal gray network,^[19]^ and right-frontoparietal network (RFPN).^[20]^ The DMN, RFPN, ECN, and left-frontoparietal network (LFPN) are related to cognition, and potentially associated with pain processing. As the insula is core regions of the SN, SN is also assumed to play a vital role in processing pain.^[21]^ Both the visual network (VN) and SMN are afferent sensory networks which are related to pain transmission.

In the study of causal relationships among brain networks, the causality model is suitable to display intranetwork communications. The Granger causality analysis (GCA) is always applied in studying causalities among brain regions or networks.^[22]^ It has been widely used in researches on stroke,^[23]^ Alzheimer's disease,^[24]^ and so on. Wang et al^[25]^ investigated the casual patterns of bilateral posterior thalamus with the rest of the brain in migraine patients by applying GCA, which found disrupted effective connection pathways between the posterior thalamus and other pain-related cortical or subcortical regions. However, to our knowledge, there is few study focused on causal relationships of pain-related brain networks in MwoA patients.

In this study, we extracted pain-related brain networks (DMN, SMN, SN, ECN, RFPN, LFPN, and VN) by using independent component analysis (ICA), and applied the multivariate Granger model to analyze the intranetwork causality in MwoA patients. We postulated that there were 2 different causal connectivity patterns in MwoA patients and healthy subjects, and significant alterations in the 7 important pain-related brain networks (DMN, SMN, SN, ECN, RFPN, LFPN, and VN) of MwoA patients in comparison with healthy subjects.

## 2 Materials and methods

This study was approved by the Dongzhimen Hospital of Beijing Ethics Committee. All subjects signed informed consents before inclusion in this study.

### 2.1 Participants

Twenty two right-handed subjects (3 males, aged 27.50 ± 5.90 years) were diagnosed as MwoA according to the classification criteria of the International Headache Society^[26]^ and met the criteria below: from 18 to 45 years old; at least 2 migraine attacks per month in the last 3 months; with at least one-year history of migraine; with no history of prophylactic or therapeutic medicine in the past 3 months; with no history of long-term use of analgesics; The exclusion criteria were as follows: other types of migraine; with history of dysmenorrhea or other chronic paining disease; with history of drug or alcohol abuse; any MRI contraindications. Another 17 healthy subjects (4 males, aged 27.18 ± 4.69 years) were recruited with no history of migraine and other neurologic disorders.

### 2.2 MRI acquisition

Images were acquired using a 3.0 Tesla MRI scanner (Siemens, Sonata Germany) at Dongzhimen Hospital, Beijing, China. Prior to scanning, all participants were asked to rest for 20 minutes and were instructed to stay still, think of nothing in particular, keep eyes closed, and not to fall asleep during scanning. Earplugs were worn to attenuate scanner noise and foam head holders were immobilized to minimize head movements during scanning.

Prior to the functional scanning, we collected high-resolution structural information for anatomical localization by using 3D MRI sequences. The resting-state fMRI data were collected using a single-shot, gradient-recalled echo-planar imaging sequence with the following parameters: repetition time = 2000 ms, echo time = 30 ms, flip angle = 90°, matrix = 64 × 64, field of view = 225 mm × 225 mm, slice thickness = 3.5 mm, gap = 1 mm, 32 interleaved axial slices, and 180 volumes.

### 2.3 Experimental paradigm

In the current research, we employed a 490-second resting scan first, and then 250-second high-resolution structural scan.

### 2.4 Data processing

The data preprocessing was conducted by software Data Processing Assistant for Resting-State fMRI (DPARSF, http://rfmri.org/ DPARSF). A total of 231 volumes for each subject were corrected for slice timing after the first 10 volumes were discarded for signal equilibrium. The slice timing and head movement were corrected for the residual phase sequence data. No subject was excluded due to excessive motion (translation > 2 mm or rotation > 2°). Functional imaging of each subject was performed based on the standardized diffeomorphic anatomical registration. The image data were normalized into the Montreal Neurological Institute (MNI) template, resampled into 3 mm × 3 mm × 3 mm, temporal band pass filtering at 0.01 to 0.1 Hz, and smoothed with a Gaussian kernel of 4 mm full width at half-maximum. Linear trends were removed from the time courses, and 8-parameter nuisance signal extraction. Finally, 9 nuisance signals (global mean, white matter, and cerebrospinal fluid signals and 6 motion parameters) were regressed out.

Independent component analysis (ICA) used the Group ICA of fMRI Toolbox (GIFT, http://mialab.mrn.org/software/gift/). After 20 times randlnit and bootstrap and regression operations, we extracted 7 resting-state networks from 3 components, which were SN, DMN, ECN, RFPN, LFPN, SMN, and VN.

Functional network connectivity (FNC, http://mialab.mrn.org/software/fnc) software was applied to analyze network causal relationships. Seven resting-state network components were selected and filtered at 0.01 to 0.10 Hz and the generalized partial directed coherence (GPDC) was selected as the measured parameter. GPDC is a linear frequency-domain quantifier of the multivariate relationship between simultaneously observed time series, which is applied in functional connectivity inference.^[27]^ Detailed introductions of the GPDC could be referred in previous paper.^[28]^

Between the 0.01 an d0.1 Hz bandwidth, the multivariate Granger model estimation was utilized. Using group level optimization from the Bayesian information criterion (BIC), the order of the model was estimated. To compare the intervention group before treatment with the control group, *P*-values were set as .05 for group comparisons. The results were corrected by false discovery rate (FDR) for multiple comparisons and final results were displayed onto a 3D standard brain surface using BrainNet Viewer.

## 3 Results

### 3.1 Demographic and clinical Information

Demographic and psychiatric characteristics of all subjects were displayed in Table 1.

**Table 1:**
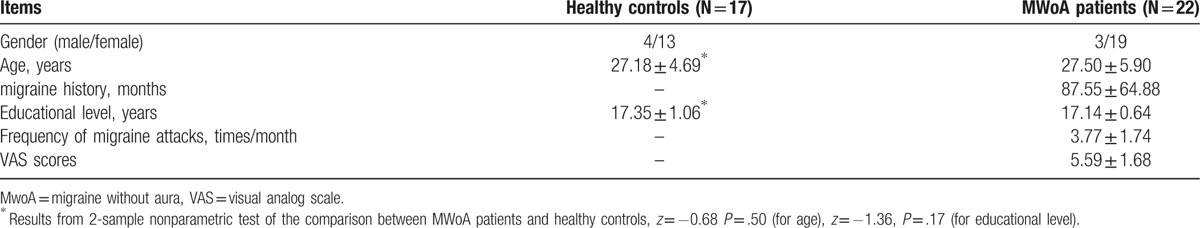
The demographic and clinical information of MWoA patients and healthy controls.

Compared with healthy subjects, MwoA patients showed no significant differences in age and educational level (*z* = −0.68, *P = .*50, for age), (*z* = −1.36, *P = .*17, for educational level). The duration of migraine ranged from 12 to 180 months (mean value, 87.55 ± 64.88 months). Frequency of migraine attacks varied from 2 to 8 times/month (mean value, 3.77 ± 1.74 times/month). Visual analog scale scores ranged from 3 to 8 (mean value, 5.59 ± 1.68).

### 3.2 ICA results

**Figure 1:**
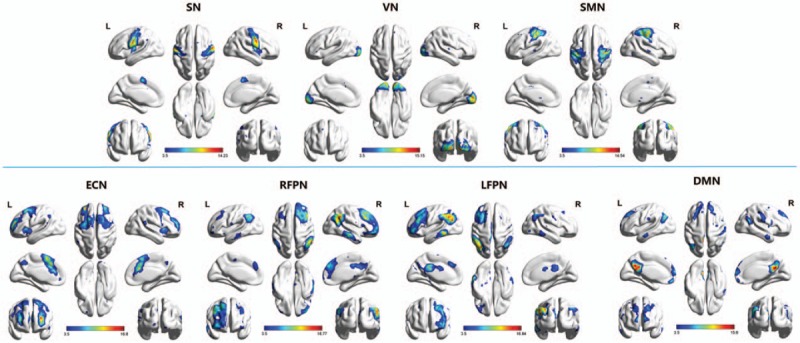
Pain-related networks screened through ICA. One-sample t test of pain-related networks of all 39 participants projected onto ICBM-152 brain surface template, as identified through ICA, including sensorimotor network (SMN), visual network (VN), default mode network (DMN), executive control network (ECN), salience network (SN), left-frontoparietal network (LFPN), and right-frontoparietal network (RFPN). The *t* value (depicted by cold to warm colors) represents the spatial statistical significance of the current networks. DMN = default mode network, ECN = executive control network, L = left, LFPN = left-frontoparietal network, R = right, RFPN = right-frontoparietal network, SMN = sensorimotor network, SN = salience network, VN = visual network.

Applying ICA in all subjects, the SN, DMN, ECN, RFPN, LFPN, SMN, and VN are extracted. Spatial positional distributions of the 7 pain-related resting-state networks are shown in Figure 1 and Table 2.

**Table 2:**
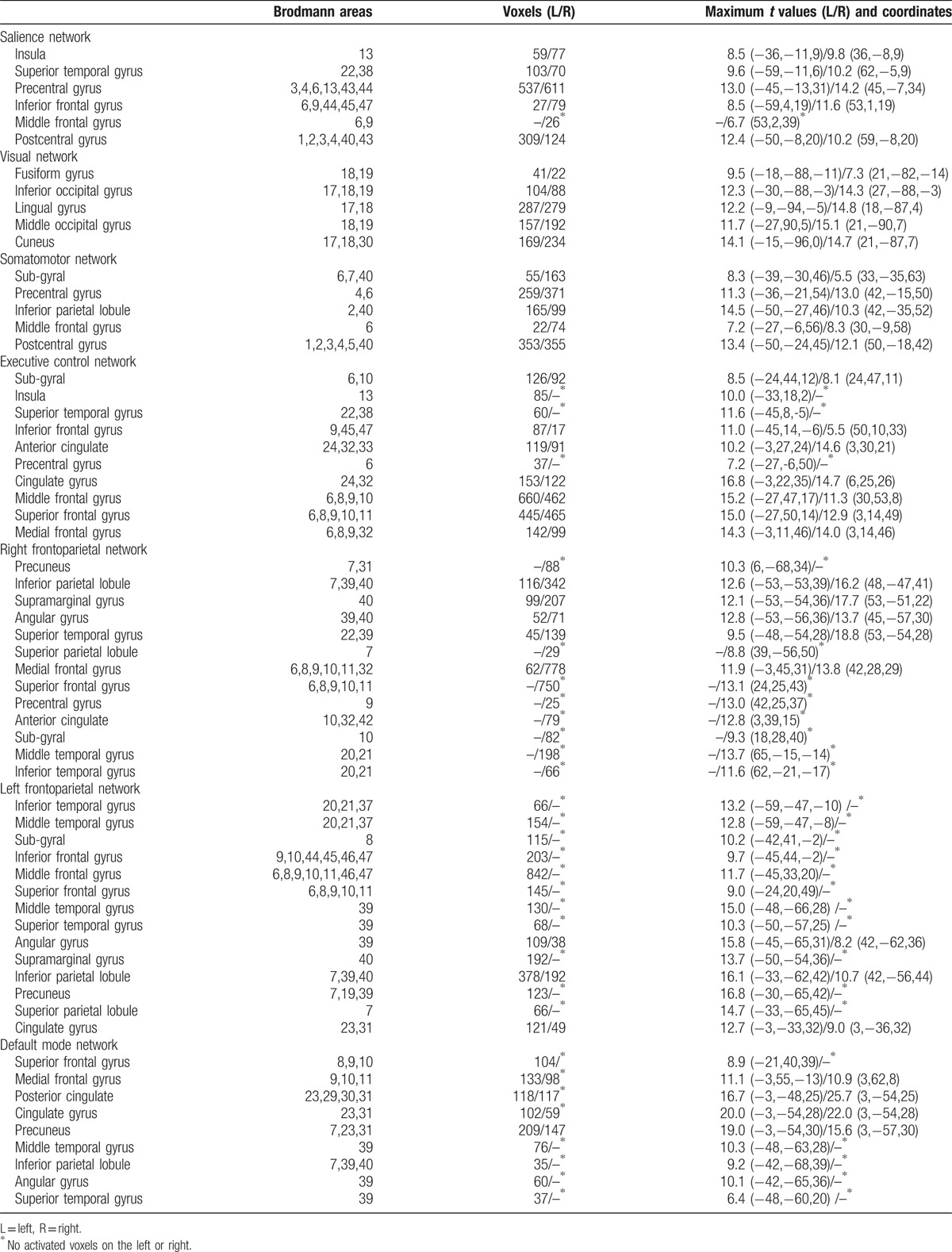
Spatial positional distributions of brain networks.

### 3.3 GCA results

**Figure 2:**
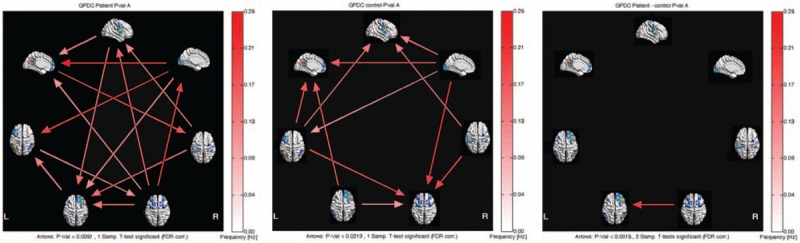
Inter- and intragroup comparisons of MwoA patients and healthy subjects. (A) one-sample *t* test result of intergroup intranetwork causal relationship of MwoA patients. (B) One-sample *t* test result of intergroup intranetwork causal relationship of healthy subjects. (C) Two-sample *t* test result of intragroup intranetwork causal relationship of MwoA patients minus healthy subjects. Panels represent visual descriptions of causal connectivity between 2 networks among the 7 resting-state networks, including sensorimotor network (SMN), visual network (VN), default mode network (DMN), executive control network (ECN), salience network (SN), left-frontoparietal network (LFPN), and right-frontoparietal network (RFPN). Arrow directions represent cause and effect. Values on the color bar (corresponding with arrow colors) demonstrate frequency at which causality was found. DMN = default mode network, ECN = executive control network, ICA = independent component analysis, L = left, LFPN = left-frontoparietal network, MwoA = migraine without aura, R = right, RFPN = right-frontoparietal network, SMN = sensorimotor network, SN = salience network, VN = visual network.

The Granger causality of the 7 pain-related resting-state brain networks in MwoA patients showed different patterns of causal connections compared with healthy subjects. MwoA patients showed more complex Granger causality connections. For the patients, RFPN and ECN were the core networks with more effective connections than others, while LFPN, VN, and ECN comprised the hubs of causal connection in healthy subjects (Fig. 2, left and center panels). Moreover, one-sample *t* test results showed that for patients, RFPN was the hub inputting information from other networks, and ECN was the hub mainly outputting information from other networks, while the RFPN and ECN were quiet opposite for healthy subjects. Two-sample *t* test results displayed that there was the significant difference between RFPN and ECN (*P* < .0015, corrected by FDR, Fig. 2, right panels).

## 4 Discussion

In the present study, we tried to investigate the difference of Granger causality connection among pain-related brain networks between MwoA patients and healthy subjects. Our results revealed that MwoA patients showed more complex Granger causality connections among pain-related brain networks than healthy subjects. More importantly, we found that only 1 significant causal relation from ECN to RFPN was observed in comparison with healthy subjects.

Resting-state networks reflect spontaneous fluctuations in the brain with the subject at rest, which could be detected by fMRI.^[29]^ Numerous researches have revealed FC abnormalities in MwoA patients, which mainly covered the pain-processing networks.^[30]^ Neuroimaging reviews also indicated that abnormalities of pain-related resting-state networks were caused by long-term ongoing pain attacks, and positively correlated with the duration of migraine.^[31,32]^ In the present study, we extracted 7 resting-state networks, which had been demonstrated to be related with pain processing. They were divided into afferent sensory networks and cognitive implementation networks. Afferent sensory networks mainly involved in pain transmission, while cognitive implementation networks mainly participated in pain processing. We also applied the GCA to detect causal influence and flow of information among the 7 resting-state networks. An interesting finding was that MwoA patients showed more complex causal connections than healthy subjects, which was in line with other chronic paining diseases, such as chronic neck pain.^[33]^ We speculated that the altered Granger causality connection among pain-related networks for MwoA patients was associated with nociceptive signals induced by frequent migraine attacks.

Another finding was that the flows of information for RFPN and ECN in MwoA patients were completely opposite to healthy subjects. Moreover, in comparison with healthy subjects, patients showed the significant difference in causal connections between RFPN and ECN. As known to all, the ECN plays an important role in cognitive processing for working memory and attention, which mainly covers dorsolateral prefrontal cortex (DLPFC).^[34]^ DMN is considered as the key component in top-down modulation functions on the cognitive and sensory aspects of psychological activities.^[35]^ Meanwhile the RFPN is involved in cognitive control and top-down modulation.^[36]^ It is confirmed that the ECN acts as a feasible modulator between DMN and RFPN,^[37]^ which involves the cognitive control over both emotional and nonemotional materials.^[38]^ Previous studies had revealed that there were abnormalities among DMN, ECN and RFPN in chronic pain diseases. An investigation of patients with persistent somatoform pain disorder found that compared with healthy subjects, patients showed decreased functional network connectivities (FNCs) between SMN and VN, between DMN and ECN and between SN and ECN as well as RFPN, and increased FNCs between SMN and LFPN.^[39]^ Xue et al. reported that MwoA patients had greater intrinsic connectivity between the DMN and ECN, and the greater connectivity is associated with the duration of migraine attacks. In contrast to these studies, the present study showed that the causal relation between ECN and RFPN was opposite in MwoA patients and control subjects, which suggested that an abnormal feedback between ECN and RFPN, and certainly affects pain processing in migraine.

As it is reported, chronic pain is a strong disruptor of intranetwork FC within the DMN, SN, RFPN, and ECN.^[14,15,17,20]^ These GCA findings suggest that brain functional networks may be performed interactively in encoding different aspects of pain. We speculate that these effects of MwoA on the functional networks may reveal the underlying neural mechanism that pain can be modulated by important causal links in cognitive networks.

There were also several limitations for the interpretation of the current results. Firstly, our results were only limited to MwoA patients, and it was unclear about other subtype patterns of migraine. Secondly, as a preliminary study, the sample size was small. This could be the reason that the periaqueductal gray network was not extracted by applying ICA, which could modulate pain perception.^[40]^ Further studies with larger sample size are needed in the future.

## 5 Conclusion

Our findings may provide new insights into the characterization of intrinsic causality connection among pain-related networks in migraine. Moreover, the change in Granger causality connection between RFPN and ECN may serve as a new potential biomarker for migraine.
